# Near Points of Convergence and Accommodation in a Population of University Students in Iran

**DOI:** 10.18502/jovr.v14i3.4787

**Published:** 2019-07-18

**Authors:** Hassan Hashemi, Mojgan Pakbin, Babak Ali, Abbasali Yekta, Hadi Ostadimoghaddam, Amir Asharlous, Mohammadreza Aghamirsalim, Mehdi Khabazkhoob

**Affiliations:** ^1^Noor Research Center for Ophthalmic Epidemiology, Noor Eye Hospital, Tehran, Iran; ^2^Noor Ophthalmology Research Center, Noor Eye Hospital, Tehran, Iran; ^3^Research and Technology Deputy, Tehran University of Medical Sciences, Tehran, Iran; ^4^Department of Optometry, School of Paramedical Sciences, Mashhad University of Medical Sciences, Mashhad, Iran; ^5^Refractive Errors Research Center, Mashhad University of Medical Sciences, Mashhad, Iran; ^6^Eye Research Center, Tehran University of Medical Sciences, Tehran, Iran; ^7^Department of Medical Surgical Nursing, School of Nursing and Midwifery, Shahid Beheshti University of Medical Sciences, Tehran, Iran

**Keywords:** Cross-sectional Study, Distribution, Near Point of Accommodation, Near Point of Convergence

## Abstract

**Purpose:**

To determine the distribution of the near point of convergence (NPC) and near point of accommodation (NPA) in a young student population in Iran.

**Methods:**

The subjects were selected using a cluster sampling method. All students underwent optometry tests, including visual acuity measurement, refraction, and cover test, as well as ophthalmic examinations. The NPC and NPA were measured using an accommodative target (near Snellen chart).

**Results:**

Of 1,595 students, the data of 1,357 were analyzed. The mean NPC and NPA in the total sample were 7.25 cm (95% confidence interval [CI], 7.02 to 7.48) and 9.99 cm (95% CI, 9.69 to 10.29), respectively. Older age was associated with an increase in the NPC, which increased from 6.98 cm in 18–20 years olds to 9.51 cm in those over 30 years. The NPA was significantly associated with age and refractive errors in the multiple linear regression model, increasing from 9.92 cm in 18–20 years olds to 11.44 cm in those over 30 years (P = 0.003). Hyperopic eyes had lower NPA than myopic and emmetropic eyes (P = 0.001). In younger age groups, the mean accommodation amplitude was lower than the mean Hofstetter value. Moreover, with age, especially after 30 years, the mean values surpassed those determined using the Hofstetter formula.

**Conclusion:**

The NPC values in this study were lower than those previously reported for identical age groups. The Hofstetter formula is not always an accurate predictor of the accommodation amplitude in the Iranian adult population.

##  INTRODUCTION

Non-strabismic vergence dysfunctions and accommodation anomalies are the main causes of symptoms such as occasional double vision, headaches, and blurred vision after prolonged near work.^[1,2,3]^ Students experience such symptoms more often than others do because of their higher demand for near vision, and this can affect their academic activities.^[1]^ The high prevalence of accommodative and binocular dysfunctions, with estimates ranging between 13.5% and 42%, point to the importance of examining the binocular vision status during routine ophthalmic examinations.^[4,5,6,7]^ However, this variation could also arise because of the variable definitions and diagnostic criteria used by different studies.

Convergence insufficiency (CI) is the most common vergence anomaly with a wide prevalence ranging from 0.8% to 13% in some studies.^[4,5,6,7][8][9][10]^ The near point of convergence (NPC) is an indicator for the diagnosis of CI and is a good measure for differentiating symptomatic from asymptomatic cases.^[11]^ Therefore, an analysis of the NPC can be essential for people with high near task demand.^[11]^ Many studies have shown that the NPC increases with age,^[12,13,14,15]^ and some indicate that the NPC is higher in men than in women,^[15]^ but its relationship with sex remains unclear.

Some studies reported a relationship between heterophoria and refractive error,^[16,17]^ while another claimed that the NPC was not related to refractive error.^[15]^ Accurate interpretation of NPC measurements requires their comparison with normal values, which may vary from one population to another.

Accommodation insufficiency (AI), a situation in which the accommodation amplitude (AA) is lower than expected for a person's age, is one of the most common accommodation dysfunctions leading to symptoms resulting from reading and near work.^[18,19]^ The measurement of the near point of accommodation (NPA) provides an index for determining the AA.

Different opinions exist about the relationship between the AA and refractive error. While some studies reported better AAs in myopes,^[20,21,22]^ other studies found no association between refractive error and the AA.^[23]^ Moreover, studies on the NPC and NPA have reported different values. The variations in the values obtained from these studies can be attributed to differences in diagnostic methods, age, refractive errors, and race in the different studies.^[9,13,24,25,26,27]^ In addition, they all examined small sample sizes, and their results cannot be generalized to other populations.

Therefore, the present study was designed to provide the NPC and NPA values in a large population of students in southern Iran and to investigate their relationship with age, sex, and degree of refractive error.

##  METHODS

This cross-sectional study was conducted in 2016-2017 in Kazerun, a city in Fars province in southern Iran. The target population of the study was all students enrolled at the four universities in Kazerun at the time of the study. The Ethics Committee of Mashhad University of Medical Sciences approved the study protocol, which adhered to the tenets of the Declaration of Helsinki. Informed consent was obtained from all participants. The students were assured that the data were anonymous and confidential. Sample selection involved a multistage stratified random cluster sampling approach, wherein each university was considered a stratum. After listing all the academic majors in each university, each major was considered a cluster, and 27 majors were randomly selected as the target clusters. Thereafter, in each university, the roster of all students studying in each selected major was prepared, and a certain number of students, proportionate to the total size, was randomly selected from each major. Finally, 1,462 students were invited to participate in the study.

All examinations were conducted at each university site in a room under daylight condition. All students underwent complete vision tests performed by an expert optometrist and slit-lamp ophthalmic examinations performed by an experienced ophthalmologist. Vision tests included the measurement of refraction with the Topcon AR 8800 auto-refractometer (Topcon Corporation, Tokyo, Japan), followed by uncorrected visual acuity measurement using a logMAR chart and manifest refraction measurement by using retinoscopy. Eventually, subjective refraction tests were performed using trial frames and ophthalmic lenses on the basis of the retinoscopy findings to determine the best-corrected visual acuity.

Binocular vision tests included the cover test, measurement of the AA and NPC, and depth perception. The AA and NPC were measured using the push-up method and an Astron Accommodative Rule (Gulden Ophthalmics, Elkins Park, PA).^[28]^

The NPA was measured using the best correction in place. While the participants focused monocularly on the “E” one line above their near visual acuity threshold, the near Snellen E chart was gradually moved toward them until they reported that the letters were blurry, and they were no longer able to maintain a clear image. At this point of sustained blur, the distance between the target and the spectacle plane was measured in centimeters. The NPA was measured three times, and the average of these measurements was recorded. The AA was calculated monocularly for each eye. The next step was NPC measurement, which was performed with the best-corrected vision in place using an Astron Accommodative Rule. This tool has a movable target and a rod marked in centimeters. A single column of letters equivalent to the 20/30 line was used at 40 cm as the target. The target was gradually moved towards the participants at a speed of 1 cm/s until they were no longer able to maintain a single image and reported double vision, or the examiner noted ocular divergence. The distance from the target to the spectacle plane was recorded as the NPC distance in centimeters. To increase measurement accuracy, NPC measurements were performed three times for each individual, and the average of the three measurements was recorded as the final NPC.

Refractive errors in this study were measured on the basis of the spherical equivalent (SE). An SE refraction worse than 0.5 diopters was defined as hyperopia, and an SE of less than -0.5 diopters was defined as myopia. Individuals with a history of eye surgery and those with a diagnosis of tropia or amblyopia were excluded from the present study.

###  Statistical Analysis

In this study, the NPC and AA in centimeters were summarized as means with 95% confidence intervals (CIs). Linear regression was used to explore the relationship of these indices with age and sex. Analysis of variance was used to determine the differences between these indices by using refractive errors. Finally, the relationship between each of these indices with age, sex, and refractive errors was examined in a multiple linear regression model. In this model, refractive errors were entered into the model by considering individuals with emmetropia as the reference group. The cluster effect was considered for more accurate estimation of the standard error. A significance level of 0.05 was considered in all the analyses. Given the higher participation of female students, weighting for sex was applied.

##  RESULTS

Of the 1,595 selected individuals, 1,462 participated in the study (91.66% participation rate). After applying the exclusion criteria (history of surgery, heterotropia, and amblyopia), a total of 1,357 individuals were analyzed in this study; of these, 1,008 (74.3%) were female. The age of the included students ranged from 18 to 39 years, and the mean age was 22.71 ± 3.0 years. The average SE ± standard deviation was -0.73 ± 1.22 diopters (-10.0 to +2.50).

**Figure 1:**
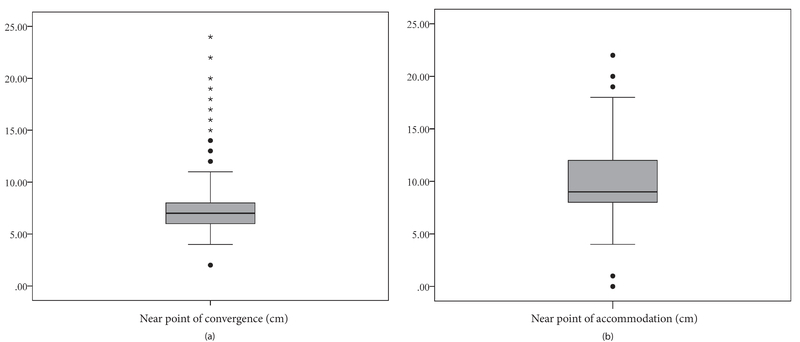
(a) The distribution of the near point of convergence (b) and near point of accommodation in university students.

Figure 1 demonstrates the distribution of the NPC and NPA in this study using a boxplot. Table 1 shows the mean NPC by age and sex. The findings of the present study showed that the mean NPC for all the students was 7.25 (95% CI, 7.02 to 7.48 cm). The mean NPC was significantly higher in male students than in female students (P = 0.046). As shown in Table 1, the NPC increased from 6.98 cm in 18–20 years olds to 9.51 cm in those over 30 years. Linear regression showed that a 1-year increase in age was associated with a 0.11 cm increase in the NPC (P = 0.005). The mean NPC in the emmetropic, myopic, and hyperopic groups was 7.29 (95% CI, 7.04 to 7.54), 7.21 (95% CI, 6.89 to 7.52), and 7.16 (95% CI, 5.6 to 8.73) cm, respectively, without any significant differences between the three groups in terms of the NPC (P = 0.322). In the multiple linear regression model, after entering the variables of age, sex, and refractive error in the model, only age maintained a significant relationship with the NPC.

**Table 1:** The mean near point of convergence and near point of accommodation


	*n*	**NPC (95% CI)**	**NPA (95% CI)**
	Total	1357	7.25 (7.02–7.48)	9.99 (9.69–10.29)
Gender	Female	1008	7.12 (6.90–7.34)	9.95 (9.57–10.34)
	Male	349	7.52 (7.14–7.89)	10.05 (9.73–10.38)
Age (years)	18–20	214	6.98 (6.69–7.27)	9.92 (9.59–10.25)
	20–25	989	7.18 (6.90–7.46)	9.92 (9.56–10.29)
	26–30	115	7.51 (7.09–7.92)	10.10 (9.55–10.65)
	> 30	39	9.51 (7.41–11.60)	11.44 (10.45–12.44)
	
	
CI, confidence interval; n, number; NPA, near point of accommodation; NPC, near point of convergence				

**Figure 2:**
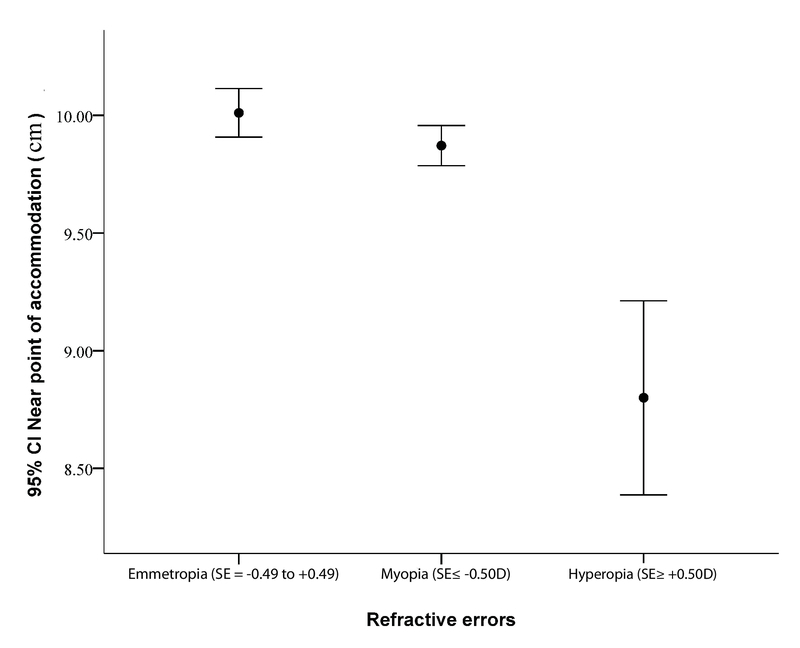
The near point of accommodation in different refractive errors. SE, spherical equivalent

The NPA was measured separately in each eye. Owing to the correlation (Pearson correlation = 0.983) of both eyes, the right eye was analyzed. The mean NPA in the present population was 9.99 (95% CI, 9.69 to 10.29 cm). As shown in Table 1, no significant difference in the NPA was observed between male and female students (P = 0.637). The NPA increased from 9.92 cm in 18–20 years olds to 11.44 cm in those over 30 years (P = 0.003). As shown in Figure 2, the NPA was significantly different among the refractive error groups, with hyperopes having the lowest NPA (P = 0.001). In the multiple linear regression model, older age was found to be associated with an increase in the NPA (coefficient = 0.09). Moreover, in this model, hyperopes had a lower NPA than did the emmetropes (P < 0.001).

##  DISCUSSION

This study evaluated the AA and convergence in a young-to-middle-aged student population in a southern city. According to the findings of this study, the mean NPC is approximately similar to the values reported by Ostadimoghaddam et al^[15]^ for the 20–29 years age range (7.59 cm). However, it is more proximal than the levels reported for the 19–30 years age group in other studies.^[11,13]^ Conversely, some studies reported a mean NPC lower than that reported in our study. Ovenseri-Ogbomo et al^[2]^ and Yekta et al^[14]^ reached the values of 6 and 5.27 cm for the 15–28 years and 18–35 years age groups, respectively. Table 2 summarizes the NPC values reported in the different studies. These different results can be attributed to several factors, including the type of target used in the NPC measurement, differences in the characteristics of the studied populations, and variations in the source and method of measurement. In the study by Momeni-Moghaddam et al,^[11]^ the most important reason for the more distant NPC (9.50 cm) than in the present study (7.25 cm) was the difference in the measurement method. In their study, the NPC was defined as the distance between the break point and the plane of the lateral canthus, while in our study, the measured distance was from the break point to the spectacle plane. Considering a 12 to 15 mm vertex distance for the glasses, a 22 mm difference between the results is reasonable. The type of target is also an important factor in NPC evaluation. It is suggested that accommodative targets result in a more proximal NPC and underestimate the NPC values. Therefore, attention to clarity may interfere with convergence and may lead to a more proximal NPC.

**Table 2:** The near point of convergence values reported by various studies


**Author**	**Sample size**		**Age (year)**	**Mean NPC (cm)**	**Target**
Momeni-moghadam et al^[11]^	124		19–24	9.50	Accommodative
Abraham et al^[13]^	150		Accommodative
	50	1–18	7.17	
	50	19–27	8.59	
	50	28–35	9.52	
Hashemi et al^[9]^	219		10–69	8.36	Accommodative
Ostadimoghaddam et al^[15]^	2,433		10–86	8.59	Accommodative
Present study	1,357		18–39	7.25	Accommodative
Larsson et al^[24]^	217		10	6.2	Non-accommodative
Ovenseri-ogbomo et al^[2]^	212		15–28	6	-
Yekta et al^[14]^	382		18–35	5.27	Accommodative
Yekta et al^[12]^	3,701		4–6	5.10	Accommodative
Scheiman et al^[27]^	175		22–37	5	Accommodative/penlight with red green glasses
Hussaindeen et al^[25]^	920		7–17	3	Accommodative
	
	
NPC, near point of convergence				

Siderov et al^[29]^ measured the NPC in 20–85 years old subjects using several types of targets and found that the NPC results were influenced by the target type only in younger individuals but not in presbyopic individuals. Thus, we can conclude that the NPC is related to accommodation. This is not unexpected because in NPC measurement, in fact, the absolute convergence is being evaluated, which is the combination of tonic, accommodative, proximal, and reflexive convergences. Therefore, stimulating accommodation increases the total amount of absolute convergence by increasing accommodative convergence, and ultimately, the measured NPC is underestimated; however, this does not occur with non-accommodative targets. Regarding the measurement method, it has been suggested that targets that are mounted on a rule provide a higher estimated NPC than targets that are moved manually and freely.^[30]^

The findings of the present study indicate an increase in the NPC values with age. Larsson et al^[24]^ and Yekta et al^[12]^ observed lower NPC values in children (younger ages). Studies have also shown that the NPC recedes with aging.^[25]^ This can be due to accommodation reduction with age, which results in reduced accommodative convergence and, consequently, a remote NPC.^[12,14,15]^

Studies on the relationship between sex and the NPC suggested a more distant NPC in men^[15]^ and boys,^[12]^ even though the inter-sex difference in the NPC in both studies was ≤ 0.1 cm, which was clinically insignificant. However, in our study, the mean NPC in men was 0.37 cm further than that in women.

We evaluated the NPA in addition to the NPC. Given the students' greater need for vision, especially near vision,^[1]^ the AA is a key factor in the rate of ocular symptoms resulting from prolonged near work. The AA is determined by measuring the NPA, which provides an estimation of the AA. Studies have shown that the AA decreases with age,^[13,14,26]^ and our results concur with their findings. The present study showed that the NPA increases with age, and thus, the AA decreases. Table 3 summarizes the AAs in different studies.

**Table 3:** Amplitude of accommodation reported by different studies


**Author**	**Sample size**	**Age (year)**	**AA (D)**	**Target**
Larsson et al${}^{\left[24\right]}$	217	10	12	Push-up/RAF rule
Hashemi et al${}^{\left[26\right]}$	1,070	11–17	11.53	RAF rule
Yekta et al${}^{\left[14\right]}$	382	18–35	11.14	Push-up/accommodative target
Hussaindeen et al${}^{\left[25\right]}$	920	11–17	11	Minus lens
Present study	1,357	18–39	9.99	Push-up/accommodative target
Abraham et al${}^{\left[13\right]}$	50	10–18	10.02	Minus lens
	19–27	9.04	
	28–35	7.34	
	
	
The amount of near point of accommodation was divided by 100 to convert it to the amplitude of accommodation in diopters.				
AA, amplitude of accommodation; D, diopter; RAF, Royal Air Force				

Most studies evaluated the AA in people under 18 years of age and reported higher values (more proximal NPA) than in the present study.^[24,25,26]^ This can be due to active accommodation in children and adolescents. The estimation of Abraham et al^[13]^ in 19–27-year-old Indians was 1.0 diopter less than that measured in our study. Rambo and Sangal^[31]^ posited the possible effect of geographic region on the AA and suggested that people residing in tropical climates tended to have a lower AA than the Europeans. However, it must be noted that their population sample was presbyopic.

**Figure 3:**
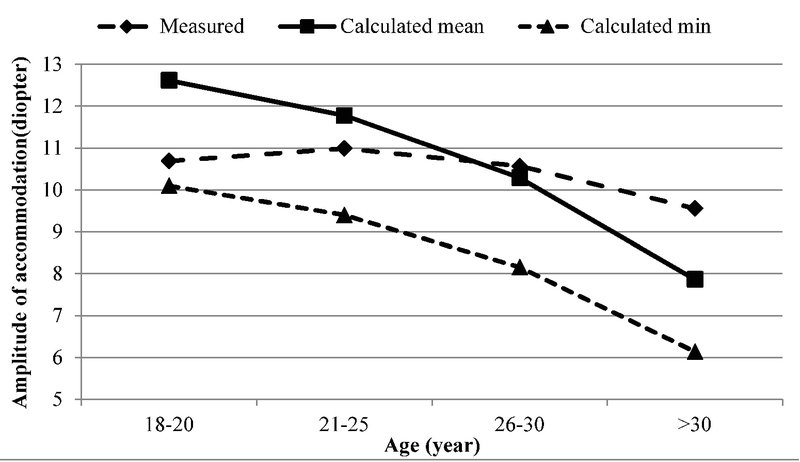
The mean and minimum amplitudes of accommodation calculated by Hofstetter's formula according to age and mean amplitude of accommodation measured in the present study.

A comparison of the AA in this study with the calculated values based on the Hofstetter formula shows that the latter is not an accurate predictor for the adult Iranian population. As demonstrated in Figure 3, the mean AA in our younger age groups was lower than the mean estimated by Hofstetter^[32]^ and was significantly higher in the older age groups, especially after 30 years of age. This formula fits the studied population best at the age of 30 years; therefore, Hofstetter's formulas, which are widely used in the diagnosis of AI, may lead to false negative results in individuals under 30 years of age and false positive results in individuals over 30 years of age.

Results regarding the relationship between the AA and sex are inconsistent. In agreement with the study by Castagno et al,^[33]^ our study did not show a significant difference in the NPA between women and men. However, Hashemi et al^[26]^ showed that the AA in 17-11-year-old girls was higher than that of boys in the same age group. They attributed the difference to factors such as education and nutrition. Yekta et al, who studied presbyopic individuals, estimated a higher AA in women and suggested that hormonal factors might have a role, especially in post-menopausal women.^[34]^

While some studies state that refractive errors do not correlate with the AA,^[23]^ Abraham et al^[20]^ reported higher AAs in myopes. This difference was observed in 35–44-year-old individuals, and no difference was observed between the types of refractive error after the age of 44 years. Maheshwari et al reported similar results.^[21]^ Charman argued that myopic eyes have weaker sympathetic innervation and stronger parasympathetic innervation than the hyperopic eyes, and therefore, when corrected with a negative lens, they have higher AAs than the hyperopic eyes.^[22]^

Investigating the relationship between the NPA and refractive errors in the present study showed a low level of NPA in the hyperopic group. Despite the greater accommodation demand with hyperopic spectacle correction than with myopic and emmetropic correction, we found a lower NPA in hyperopic patients. There are several possible reasons for this finding. First, hyperopes may go without glasses, especially at younger ages. Therefore, they use their accommodation for near work and even far vision more than emmetropic and myopic individuals do.^[35]^ Hence, the frequent use of accommodation may contribute to better development of accommodation and a stronger accommodation reflex. This is similar to orthoptic reinforcement exercises that increase the amplitude with repeated accommodation,^[36,37]^ which occurs naturally in hyperopic eyes. Another reason may be the magnification effect of glasses.^[38]^ The NPA was measured with optical correction in place, and the refractive correction of hyperopia was achieved using convex lenses, which magnify the image. The blur caused by defocus is understood much later in the larger retinal images because the details in a larger image are more recognizable than those in a smaller one. Thus, the patients may report the blur much later.^[39,40]^ Hyperopes may also report blur relatively later with the push-up method, and thus the NPA may be underestimated.

This study reports normative values in large university students using a valid method; however, it has some limitations. Cycloplegic refraction was not performed. In addition, similar to other studies, refractive errors were measured based on SE that may affect the interpretation of results in some cases such as mixed astigmatism. Moreover, the age range of participants was relatively wide.

In conclusion, this study estimated the NPC and NPA values in a large population of university students in southern Iran. The NPC values were lower than those reported in most other studies on this age group. Clinicians should be aware of the impact of age and sex during the evaluation of CI symptoms and other binocular vision dysfunctions. The discord with the Hofstetter formula also should be considered in the diagnosis of AI.

##  Financial Support and Sponsorship

Nil.

##  Conflicts of Interest

There are no conflicts of interest.
